# Comparing Methods of Seizure Response Monitoring During Electroconvulsive Therapy: Comparer les méthodes de surveillance des interventions en cas de crise durant la thérapie par électrochocs

**DOI:** 10.1177/07067437231223340

**Published:** 2023-12-28

**Authors:** Timothy Hierlihy, Gerald Mugford, Weldon Bonnell, Mohamed A. Mekawy

**Affiliations:** 1 Faculty of Medicine, Discipline of Psychiatry, 7512 Memorial University of Newfoundland, St. John's, Canada

**Keywords:** electroconvulsive therapy (ECT), electroconvulsive shock, electroencephalogram (EEG), electroencephalography, seizure, ECT, choc électroconvulsif, thérapie par électrochocs, EEG, électro-encéphalogramme, électro-encéphalographie, crise

## Abstract

**Objective:**

Electroconvulsive therapy (ECT) is used to treat several mental illnesses. Seizure duration is used to determine if the administered stimulus was adequate. Duration is estimated by electroencephalogram (EEG) interpretation and/or observed motor response (OMR). Neither method is considered the gold standard. This study investigated the relationship between the 2 methods. The hypothesis was that both EEG and OMR would be significantly positively correlated. Previous researchers have suggested that the 2 methods resulted in different estimates.

**Methods:**

A case series was conducted using recorded estimates obtained prospectively from 102 ECTs on adult psychiatric inpatients.

**Results:**

A strong positive association was not observed in this study, correlation coefficient 0.510 (*p* < 0.001).

**Conclusions:**

This study suggests that the 2 methods differ, and further research is needed to determine the best indicator of adequate treatment.

## Abrégé

**Objectif:**

La thérapie par électrochocs (ECT) est indiquée dans plusieurs maladies mentales. La durée de la crise est utilisée pour déterminer si le stimulus administré était adéquat. La durée est estimée par l’interprétation de l’électro-encéphalogramme (EEG) et/ou de la réponse motrice observée (OMR). Aucune des deux méthodes n’est considérée l’étalon-or. La présente étude a investigué la relation entre les deux méthodes. L’hypothèse avançait que l’EEG et l’OMR seraient significativement et positivement corrélées. Les recherches antérieures ont suggéré que les deux méthodes résultaient en des estimations différentes.

**Méthodes:**

Une série de cas a été menée à l’aide d’estimations enregistrées obtenues prospectivement de 102 ECT sur des patients adultes psychiatriques hospitalisés.

**Résultats:**

Une forte association positive n’a pas été observée dans cette étude.

**Conclusions:**

La présente étude suggère que les deux méthodes diffèrent, et qu’il faut plus de recherche pour déterminer le meilleur indicateur du traitement adéquat.

## Introduction

Research has demonstrated electroconvulsive therapy's (ECT's) efficacy in treating various mental illnesses.^1^ Seizure duration estimation is used to determine if the stimulus administered resulted in an adequate response.^1^ If a response is considered to be inadequate, the stimulus parameters are changed. The 2 most common methods to estimate seizure duration are clinical observation of the motor seizure response, or electroencephalogram (EEG) to indirectly measure electrical activity in the brain. Both methods are indirect measures of electrical activity within the brain. If they differ, then they cannot be the best measure of adequate treatment from a clinical perspective.

There are no clear North American guidelines on the exact seizure duration considered being adequate, or which method of measuring seizure duration is best. In the UK, the NICE guideline on depression references the Handbook on ECT published by the Royal College of Psychiatrists for current standards on ECT.^2^ This handbook recommends using EEG monitoring to assess qualitative aspects of a seizure as opposed to duration alone. The authors state that observed motor response (OMR) can inform this assessment.^3^ The Canadian Psychiatric Association (CPA) recommends a minimum seizure of 15 s as measured by EEG. They recommend using both EEG and motor response for monitoring during treatment.^1^ The American Psychiatric Association (APA) recommendations on the practice of ECT state that when seizure duration is <15 s in both motor and EEG manifestations, the likelihood is high that the seizure was limited by insufficient electrical stimulation and that the treatment was inadequate. They described the motor method as the simplest and most reliable. However, they stated that EEG monitoring should also be used. The reasons given include that occasionally patients may have adequate seizures without motor manifestations, EEG seizures are commonly of longer duration than motor movements, and rarely patients may have prolonged seizures or return of seizure activity that does not manifest motor movements.^4^ The CPA references the APA in their recommendations, and the APA did not provide a specific reference for their recommendation.^1,4^

Considering the potential effect of muscle relaxants on motor output, it would seem plausible that EEG would be the more sensitive and robust measure of seizure activity. However, both the CPA and APA recommend using both methods. This apparent redundancy inspired the research question that was the focus of this study.

Given the lack of clear guidelines on which estimation method to use, the literature was reviewed with the following research question in mind: Is there a significant difference in the duration of monitored seizure during ECT when measured by OMR versus EEG monitoring?

The articles reviewed used variable techniques for estimating seizure duration and reported varying results. The absence of a gold standard for seizure quantification during ECT may result in suboptimal care. If seizure duration correlates to treatment efficacy (not the focus of this study), an accurate method of measuring seizure duration is warranted.

This study set out to quantify the correlation between 2 methods of seizure duration estimation. The hypothesis was that there would be a strong positive correlation between OMR and EEG interpretation when used to estimate seizure duration in response to ECT stimulus.

## Methods

The study was designed as a case series. A single group of patients received the same 2 investigations.

The study population was Psychiatry Inpatients (≥18 years) at the Health Sciences Centre, a tertiary care centre in St. John's, Newfoundland, Canada who were receiving ECT. Any adult patient receiving ECT was included, regardless of age, gender, diagnosis, comorbid conditions, medications, adjunctive therapy, or number of ECT treatments. The reason for not having exclusions based on the previously mentioned parameters was that this study was only interested in comparing 2 methods of estimating seizure duration during treatment. A more in-depth study could investigate the correlation between seizure estimation by each method and treatment response. This would be challenging, as ECT is used to treat multiple conditions, the number of treatments administered is variable, and measuring treatment response can be subjective in nature. An alternate study design would be more appropriate if using treatment response as the primary outcome, this would require strict inclusion and exclusion criteria.

A sample size of 100 was chosen. This allowed for the detection of a correlation coefficient as small as 0.28. Although 0.28 is a weak correlation, this sample size allowed for determination of a larger correlation as well. The study required recorded OMR times and EEG interpretations from 100 ECT procedures, not from 100 separate patients.

Consent was not sought from the patients whose data was included in this research. This was made clear in the proposal for ethical approval, which was successful. The rationale behind proceeding without consent involved several reasons. First, participation in this study did not involve any alteration to the intervention patients received. Second, patients receiving ECT are often doing so involuntarily, and would therefore not be capable of giving consent. Finally, no personal information of any kind was recorded aside from the seizure durations. As such, demographic information for the 100 procedures was not collected. Data was collected from patient charts over a 4-month period. In the absence of specific demographics, program statistics were obtained for the 12-month period from which this 4-month sample was taken. A total of 305 individual ECT procedures took place in these 12 months. The average patient age was 59, with a range of 20 to 93. The proportions of females and males were 62% and 38%, respectively.

Data was collected from patient charts over a 4-month period. The 4 psychiatrists administering ECT at the study site agreed to record data for the study. Each psychiatrist was experienced with ECT at this site, and 2 of the psychiatrists received their ECT training from the other 2. Both motor response and EEG were used to estimate seizure duration. Prior to stimulus administration, a single EEG lead was applied to the patient's mastoid process. The psychiatrist observed the patient from the time of stimulus delivery until observable movement ceased. They recorded this duration and then interpreted the EEG printout produced by the ECT machine. Treatments were administered with a Spectrum Mecta Q5000 ECT machine. Stimulus duration was brief or 1 ms, and frequency ranged from 30 to 90 Hz. The majority of treatments utilized bitemporal electrode placement. The motor response was recorded before the EEG was viewed, making it independently determined. The EEG was not interpreted independently, as the psychiatrist observed the motor response time before seeing the EEG. For this reason, the EEG interpretations of the psychiatrists were not included in this dataset. To circumvent this potential source of bias, the primary investigator interpreted the EEG recordings before looking at the recorded motor response time. The EEG printout from the ECT machine was the only factor considered when estimating seizure duration via EEG, no physiological data was measured or recorded. As such, the 2 methods of seizure duration estimation were assessed independently. The primary investigator interpreted all of the EEG readings. While 4 psychiatrists determined motor durations, each individual motor duration was determined by a single psychiatrist. Two of the 4 psychiatrists trained under the other 2.

All statistical analyses were carried out using SPSS (IBM® SPSS® Statistics 2012, Version 21). The data was normally distributed. A paired *t*-test was used to compare the 2 methods. A Pearson correlation coefficient was calculated to measure the strength of their association. A linear regression analysis was used to quantify the association.

The paper “Statistical Methods for Assessing Agreement Between Two Methods of Clinical Measurement” describes an approach for assessing agreement based on graphical techniques and simple calculations.^5^ The authors highlighted several limitations of statistical techniques commonly used to assess agreement. Given that the purpose of this study was to assess the agreement between 2 methods of seizure duration estimation, this technique was well suited for the data analysis. The method described by the author supplemented the previously mentioned analysis to aid in making clinical extrapolations. The decision to include this supplemental analysis was made prior to the data collection phase.

Approvals were granted by the Health Research and Ethics Board of the province of Newfoundland and Labrador and the Research Proposal Approval Committee of Eastern Health.

## Results

No patient demographic data was collected. The CPA and APA recommendations do not offer altered seizure durations based on any clinical or demographic variables. Considering this, as well as the focus of this study on comparing 2 methods of measuring seizure activity, demographic data was not collected. Table 1 contains EEG and OMR seizure estimates obtained from 102 ECT procedures. The average seizure duration estimate was 29 s for EEG and 24 s for OMR. EEG estimates ranged from 0 to 79 s. OMR estimates ranged from 0 to 54 s. The average difference (EEG–OMR estimate) between the 2 measures was approximately 5 s. The OMR estimates ranged from 38 s longer to 42 s shorter than their respective EEG estimates.

**Table 1.** EEG and OMR Means, Standard Deviation, Range and Duration Differences.

	EEG	OMR	Difference EEG − OMR
*N*	102	102	
Mean (s)	29.08	23.68	5.4020
Standard deviation (s)	15.958	11.997	14.26153
Range (s)	0–79	0–54	

*Note*. EEG = electroencephalogram; OMR = observed motor response.

Table 2 displays the results of the paired samples *t*-test. The EEG estimation was 5 s longer than the OMR estimation with a 95% confidence interval of 3 to 8 s. The *t*-value generated by the analysis was approximately 4, indicating that the 2 methods used were different. The values were statistically significant.

**Table 2.** Paired Samples *t*-Test Comparing EEG to OMR.

	Paired differences				*t* value	Degrees of freedom	Significance
	Mean (s)	Standard deviation	Standard error mean	95% confidence interval			
EEG − OMR	5.402	4.262	0.412	0.601 to 0.203	3.825	101	<0.001

*Note*. EEG = electroencephalogram; OMR = observed motor response.

The Pearson correlation coefficient (r) is a measure of the linear correlation between 2 variables. The Pearson correlation coefficient was 0.510 (*p* < 0.001). This indicated a moderately positive linear relationship between the 2 methods of measure.

A linear regression analysis yielded an *R*2 value of 0.26. This indicates that the linear regression model explains 26% of the difference between the 2 measures. Conversely, unknown factors explain 74% of the difference between the 2 measures. The relationship between the 2 measures is not robust.

**Figure 1.**
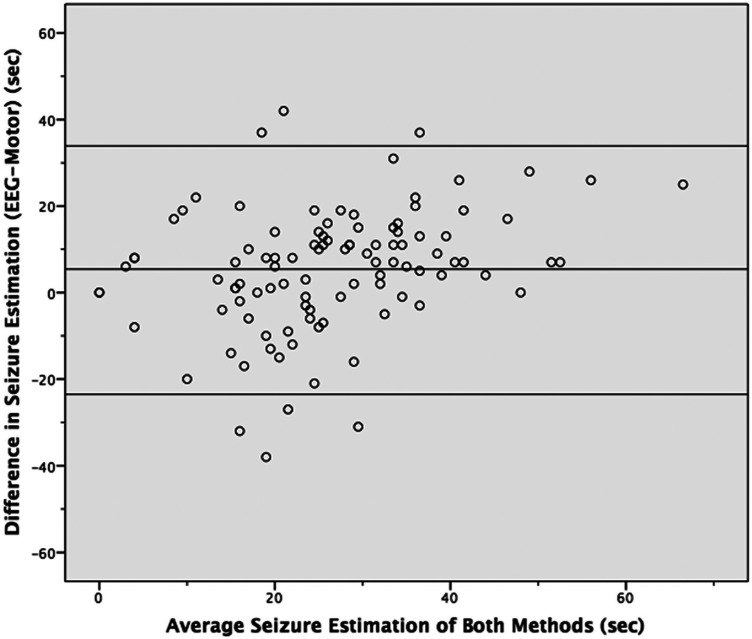
Difference against mean for seizure estimation of both methods.

Figure 1 is a scatter plot showing the average seizure estimate for each ECT procedure on the *x*-axis and the difference in seizure estimation between the 2 methods of measure (EEG-motor response) on the *y*-axis. The graph also includes 3 horizontal lines. The middle line represents the average difference between the 2 methods (5.4 s). The highest and lowest lines represent the mean plus or minus 2 standard deviations, respectively (33.4 and −22.6 s), or the 95% confidence interval. On average, one would expect the EEG estimate to be 5.4 s longer than the OMR. For any given OMR seizure estimation, one would expect that 95% of the time the corresponding EEG estimates would be anywhere from 33.4 s longer to 22.6 s shorter. This supports the previous analysis indicating that the relationship between the 2 methods of estimation was not strong.

The CPA and APA designated 15 s as the minimum adequate seizure duration.^1,4^ An average difference of 5.4 s is large when dealing with a threshold of 15 s. The range one would expect corresponding values to fall 95% of the time was large at 56 s. This suggests that the 2 methods are not closely related.

**Figure 2.**
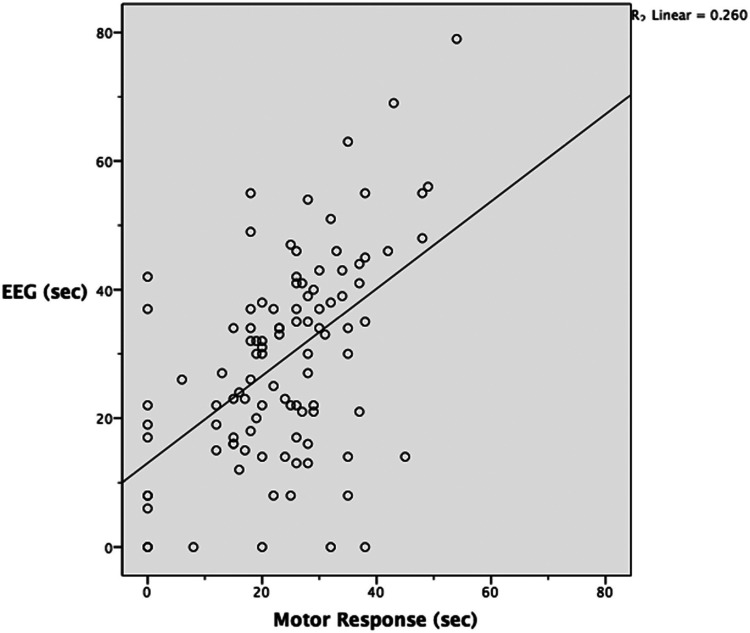
Estimated seizure duration with linear regression line included.

Figure 2 plots the EEG and OMR seizure estimates against each other, along with a linear regression line. This scatter plot includes several points that contained estimates of zero for EEG and/or OMR. Zero values indicate that the method of seizure duration estimation used did not identify any seizure activity. Multiple calculations were done to investigate the impact of these zero values. First, only points that contained an OMR of zero were excluded. Then, only points containing EEG values of zero were excluded. Finally, any point that included a zero value was excluded. The calculations included mean difference, 95% confidence interval of the mean difference, standard deviation, Pearson correlation coefficient (*r*), and the linear regression value *R*^2^. Regardless of exclusion, the variation in mean difference was 2.5 s, the Pearson correlation coefficient was consistently 0.5, and the variation in *R*^2^ was 0.05. Altering the analysis based on 0 values had little impact on the results.

The analysis was based on the continuous variable of time. Post hoc, the data was converted from continuous to categorical and analyzed. The APA and CPA suggest 15 s as the minimum adequate seizure duration.^1,4^ The seizure estimate data points were categorized into either adequate (≥15 s) or inadequate response (<15 s). The number of procedures which resulted in seizure response being deemed adequate by each measurement modality, and the amount of agreement between the 2 was then quantified. For 79 cases (77%) both EEG and OMR agreed. For 10 cases (10%), the EEG identified the response as adequate, while the motor response identified it as inadequate. For 13 cases (13%), the motor response identified the response as adequate, while the EEG response identified it as inadequate.

**Figure 3.**
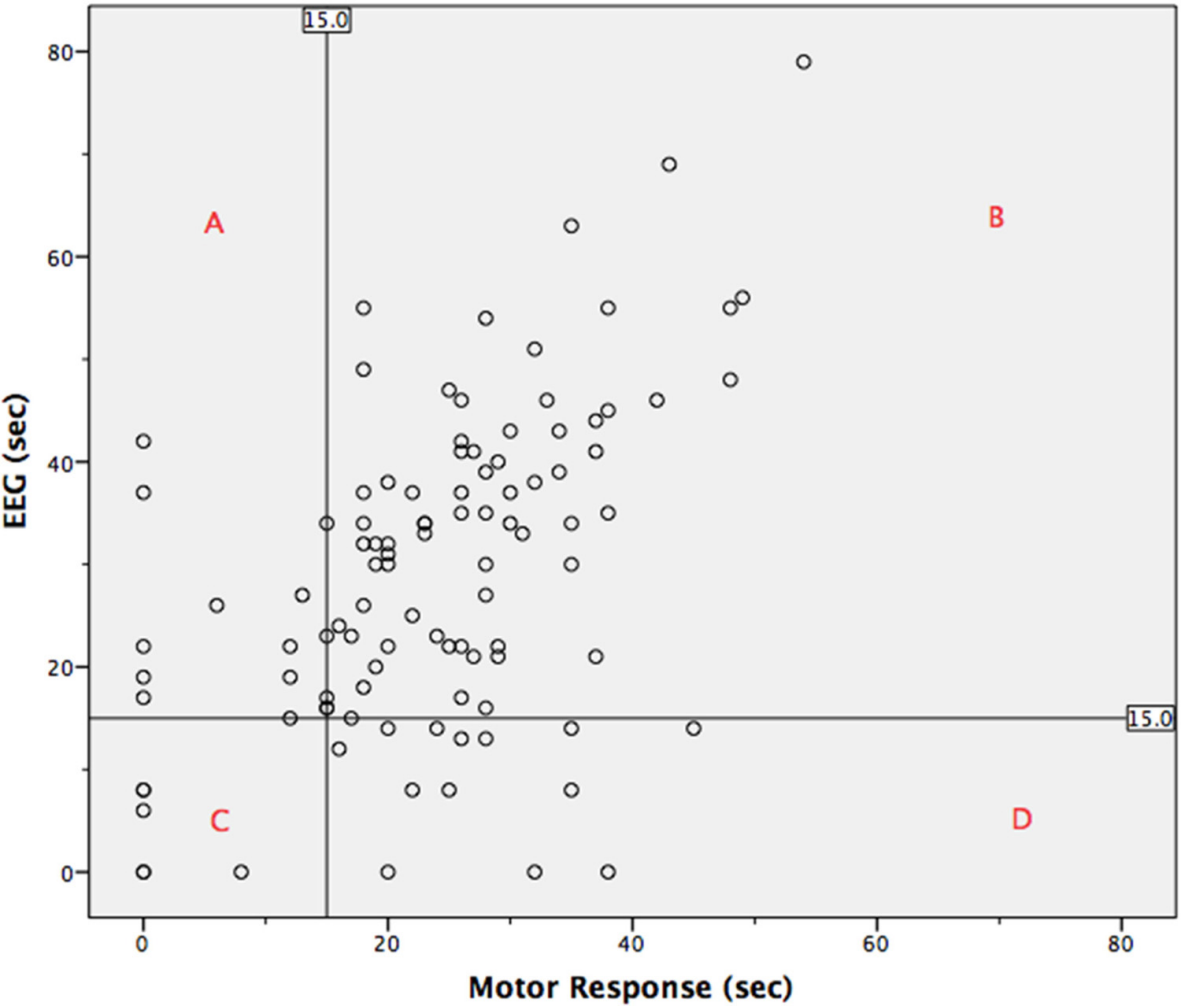
Scatter plot of electroencephalogram (EEG) and motor response including reference lines that represent the minimum duration of adequate seizure duration (15 s).

The scatter plot in Figure 3 plots the EEG and OMR seizure estimates against each other. It includes a vertical and horizontal line representing the 15 s mark for OMR and EEG response, respectively. These lines indicate the minimum seizure duration considered to be adequate. The graph has been divided into 4 quadrants by the intersection of these reference lines. Quadrant A represents the seizure responses considered to be adequate by EEG response, but not OMR (10 cases). Quadrant B represents the seizure responses considered to be adequate by both methods of estimation (73 cases). Quadrant C represents the seizure responses considered to be inadequate by both methods of estimation (6 cases). Quadrant D represents the seizure responses considered to be adequate by OMR, but not EEG response (13 cases).

Neither EEG nor OMR have been universally accepted as the gold standard for seizure duration estimation. Using 15 s as the cut-off for an adequate seizure duration, the number of ECT procedures that would have been deemed adequate can be determined for several scenarios. Requiring an adequate response in both methods would have deemed 73 (72%) treatments adequate. Using EEG alone would have deemed 83 (81%) treatments adequate. Using OMR alone would have deemed 86 (84%) treatments adequate. Using both methods and requiring an adequate response in only 1 method would have deemed 96 (94%) treatments adequate.

## Discussion

We hypothesized that there would be a strong positive correlation between OMR and EEG interpretation when used to estimate seizure duration in response to ECT stimulus. A strong positive correlation was not found. The Pearson correlation coefficient of 0.51 was moderately positive. The linear regression model can account for 26% of the difference between the 2 measures, meaning 74% of the difference can be attributed to unknown factors.

These findings were supported by the alternative analysis.^5^ While the mean difference between the methods was 5.4 s, the 95% confidence interval ranged from 33 s longer to 23 s shorter when the EEG response was compared to the motor response. This disparity is large when using 15 s as the minimum duration for an adequate response.

Converting the continuous variable of time into the categorical variables adequate or inadequate did not identify a strong correlation between the methods either. They agreed in 77% of the cases.

The EEG interpretation and motor response methods are indirect measures of seizure duration. This study was designed to quantify the relationship between the methods. The methods were found to differ.

Identifying a difference is in keeping with previous publications. Benbow et al.^6^ determined that EEG seizure estimation was longer than OMR estimation based on a retrospective analysis of 67 patients who received 95 courses of ECT, with each course ranging from 1 to 20 treatments. Mayur et al.^7^ came to similar conclusions in a prospective case series.

Previous publications have identified a difference in these methods of measure. They reported that the average seizure duration estimation based on EEG exceeded the estimates based on OMR.^6,7^ This is in keeping with the average difference found in this study. This study showed that on average EEG estimates were 5 s longer than motor estimates. However, when comparing individual pairs of estimates, the 95% confidence interval showed that EEG can be as much as 33 s longer or 23 s shorter than the motor estimate. There were many cases where OMR exceeded EEG response. The findings of this study suggest that the 2 measures differ and that there is not a strong, consistent, predictable relationship between them.

Swartz^8^ presented 2 case reports which demonstrated that the 2 estimation methods do not always agree. His findings are in keeping with the results of this study in that the 2 methods lead to different results.

It remains unclear as to which of the 2 methods of seizure duration estimation is the most accurate, or more importantly most clinically relevant for determining treatment adequacy. Given that the methods differ, both cannot be the best measure of adequate treatment from a clinical perspective. Clinical outcome is the best measure of clinical outcome. It would be challenging to determine which method best correlates to clinical outcomes. More research is needed to determine which method, if any, is the best measure of treatment adequacy.

## Conclusions

This study was carried out to quantify the relationship between 2 indirect measures of ECT stimulus-response. The original hypothesis was not supported. A strong positive correlation between EEG interpretation and OMR for seizure estimation was not observed in this study.

A post hoc analysis consisted of having the continuous variable time converted to the categorical variables of adequate (≥15 s) and inadequate (<15 s) response. This allowed for considering the agreement of the 2 measures in relation to their clinically important role of identifying adequate treatment response. The measures were found to agree for approximately three-quarters of the cases included.

It is not possible to make any recommendations on which of the 2 methods is superior based on the findings of this study. The relationship between the 2 was all that was considered, at no stage was accuracy, appropriateness, or indication of efficacy examined. This study reflects the lack of consensus as to which method is preferred, simply demonstrating that they differ.

Based on the findings of this study, as well as findings from others, further research is required to determine which method is the best indicator of seizure response, and more importantly ECT efficacy. Research of this nature would be more complicated. Establishing superiority would be best achieved by double-blinded, randomized, controlled clinical trials. In the UK, Semple et al.^3^ pointed towards assessing seizure quality over duration. The characteristics of an EEG can be used to estimate the quality of a seizure as well as its duration. This interpretation can take other factors into account, such as observed motor seizure duration.

Until such a time that a preferred method can be identified, it would be prudent to use both methods simultaneously. Ideally, the team administering ECT would collaborate with the patient's attending physician. This would allow both parties to consider clinical response as well as estimated seizure duration when making decisions regarding management.

### Limitations

A limitation of this study is its design. A case series is not the most robust form of research, but it was the most appropriate choice to answer the research question posed. Without an established gold standard, the best that can be done is analysis for correlation and agreement of measures.

Another limitation of this study design was that it was focused on comparing 2 separate indirect measures. ECT is not administered because it causes a motor response or EEG changes. ECT is administered because it is an effective treatment for multiple forms of mental illness. Estimated seizure duration in response to ECT is used to guide changes in the stimulus administered in treatments. A randomized double-blind trial comparing treatment outcomes when a motor response is used versus EEG interpretation would be the ideal way to investigate which is the superior method. Unfortunately, there are several obstacles to attempting such a trial. ECT is used to treat a myriad of mental illnesses, and patients often have comorbid psychiatric conditions. Patients often have medical comorbidities, which may include epileptiform conditions. There are often various medications involved, these can include anticonvulsants. This study was focused on quantifying the relationship between the 2 methods, no inferences can be made based on the results regarding either method or treatment response.

Another limitation of this study was the use of seizure data alone, without demographic or clinical data to provide further clinical context. While this study was focused on comparing 2 indirect measures of seizure activity, clinical and demographic data may have added to the discussion of the relationship with clinical outcome.

Another limitation of this study was its size. The 102 treatments that took place were adequate to determine a strong correlation between the measures. However, the study did not include 102 people. This means that a smaller number of people would have contributed multiple readings. The motor and EEG measurements may not be independent, and individual variability may have impacted the interpretability of the results.

The OMR seizure estimates were carried out by the 4 different psychiatrists who administered the ECT procedures. It is possible that there may have been some interrater variability which was not accounted for in this study. The EEG estimation was carried out by a single investigator, removing the potential for interrater variability. Having potential interrater variability in the motor estimation, but not the EEG may have introduced a potential bias.
